# Measuring Temporal Trends and Patterns of Inpatient Antibiotic Use in Northwest China’s Hospitals: Data from the Center for Antibacterial Surveillance, 2012–2022

**DOI:** 10.3390/antibiotics13080732

**Published:** 2024-08-05

**Authors:** Aizezijiang Aierken, Xiaochen Zhu, Ningning Wang, Jiangtao Zhang, Weibin Li, Haishaerjiang Wushouer, Kaisaier Abudukeremu

**Affiliations:** 1 Department of Pharmacy, Xinjiang Medical University, NO. 393 Xinyi Road, Urumqi 830017, China; 2 International Research Center for Medicinal Administration, Peking University, Beijing 100191, China; 3 Department of Pharmacy Administration and Clinical Pharmacy, School of Pharmaceutical Sciences, Peking University, 38 Xueyuan Road, Haidian District, Beijing 100191, China

**Keywords:** inpatient, antibiotics use, surveillance data, China

## Abstract

Background: The challenge of emerging antimicrobial resistance and variation in antibiotic use across provinces in China call for knowledge on antibiotic utilization at the regional level. This study aims to evaluate the long-term trends and patterns of antibiotic usage in Xinjiang Province, the largest provincial-level division located in the northwest of China, aiming to provide evidence in enhancing provincial antimicrobial stewardship (AMS) and developing policy measures to optimize regional antimicrobial use. Methods: This was an ecological study with temporal trend analysis on inpatient antibiotic utilization, with antibiotic use data from 92 public hospitals covered by Xinjiang’s Center for Antibacterial Surveillance from 2012 to 2022. Antibiotic use was measured by the number of daily defined doses per 100 patient days (DDDs/100 pds). Patterns of antibiotic use were described by Anatomical Therapeutic Chemical (ATC) subgroups and the Access, Watch, Reserve (AWaRe) classification. The Average Annual Percent Change (AAPC) of antibiotic use and the corresponding 95% confidence intervals (CIs) were calculated to describe the trend of antibiotic use over time. Joinpoint regression was performed using the Weighted Bayesian Information Criteria (WBIC) model with a parametric method. A pairwise comparison between secondary and tertiary hospitals was conducted to explore disparities in antibiotic use across hospital levels. The most commonly used antibiotics were also analyzed. Results: The total inpatient antibiotic use in Xinjiang was 27.6 DDDs/100 patient days in 2022, with a significant decreasing trend during 2012–2022 (AAPC, −2.0%; 95% CI, −3.6% to −0.4%). The Watch group antibiotics were the most used AWaRe category, with the Access-to-Watch ratio decreasing significantly from 46.4% to 24.4% (AAPC, −6.8%; 95% CI, −8.4% to −5.1%). No significant difference was found in the trend of total antibiotic use between secondary and tertiary hospitals, but there were disparities across hospital levels in subgroups. Third-generation cephalosporins, second-generation cephalosporins, and fluoroquinolones remained the top three antibiotic class throughout the study period. The number of antibiotics accounting for 90% of the total antibiotic use decreased from 34 antibiotics in 2012 to 18 antibiotics in 2022. Conclusions: The decreasing trend of inpatient antibiotic use in Xinjiang’s public hospitals reflects the effects of continuous AMS implementation. Patterns of antibiotic use underscore the need for further efforts on evidence-based antibiotic selection and for analyses on the appropriateness of antibiotic use.

## 1. Introduction

The challenge of antimicrobial resistance (AMR) has been an urgent global public health problem worldwide [1]. It is estimated that, in 2019, there were approximately 4.95 million deaths associated with bacterial resistance worldwide, with 1.27 million deaths directly attributable to AMR. Without proactive measures, it is projected that, by 2050, 10 million people will die annually due to AMR, potentially causing a 1.1–3.8% decline in the global annual GDP [2,3]. The irrational use of antibiotics is one of the main drivers of the rapid development of AMR [4,5,6]. In China, antibiotic misuse has been a significant issue. National monitoring of outpatient medication in secondary and tertiary hospitals indicated that, between 2014 and 2018, approximately 18.85 million antibiotic prescriptions were issued, with up to 9.69 million (51.4%) deemed inappropriate [7].

The World Health Organization (WHO) regards surveillance of antibacterial use as a cornerstone of antimicrobial stewardship [8] and has been stressing the need for strengthening the knowledge and evidence base of AMR through surveillance and research [9]. The WHO Global Antimicrobial Resistance and Use Surveillance System (GLASS) was launched in 2015, and the monitoring of antimicrobial consumption (AMC) was incorporated in 2020. The GLASS-AMC methodology has recently been developed [10,11,12] to assist countries in monitoring antimicrobial medicines at the national level [1]. As one of the first patch of lower-middle- and upper-middle-income countries (LMICs and UMICs, respectively) echoing the WHO’s endeavors, China has established a national, budgeted operational plan with monitoring networks to address AMR [13,14], including the continuous expansion of a surveillance network in terms of antibacterial use and AMR. The former Chinese health ministry established the Center for Antibacterial Surveillance (CAS) in 2005 and has been making continuous efforts in improving the coverage and quality of surveillance [15]. By 2021, it covered 2694 tertiary hospitals and 4100 secondary hospitals, which accounted for 90% and 40% of all these hospitals, respectively, in mainland China [16,17,18]. The methodology for CAS surveillance is based on the Anatomical Therapeutic Chemical (ATC) classification system and the defined daily doses (DDDs) methodology [15], which is consistent with the methodology of the GLASS-AMC surveillance framework, thus setting a solid foundation to generate quality representative data to inform national policy development, evaluate trends, and allow for comparison across countries [1].

A previous study [15] found that there was a considerable variation in the trend of antibiotic use between provinces in China, which warrants further studies on regional data to support provincial antibiotic stewardship. However, there has been relatively fewer published regional studies focused on underdeveloped remote regions of China. Current studies have been characterized either by limited sample hospitals [19] or procurement data analysis due to the limited usage data accessibility [20].

Thus, to provide tailored region-level support to optimize regional antimicrobial use, it is necessary to evaluate up-to-date antibiotic usage over a long-time span at the province level to reveal long-term trends and patterns of regional antibiotic utilization. Therefore, to provide guidance in designing evidence-based policy measures in further strengthening antibiotic stewardship at the regional level, this study aims to assess 11-year trends and patterns in inpatient antibiotic use in Xinjiang’s public hospitals.

## 2. Results

### 2.1. Total Antibiotic Usage

**Figure 1:**
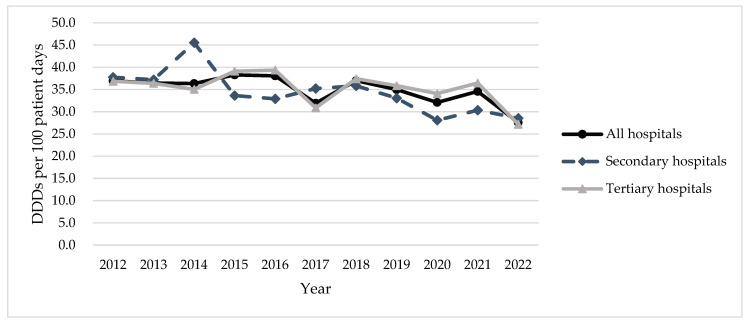
Total inpatient antibiotic use in secondary and tertiary hospitals in Xinjiang, 2012–2022.

The number of hospitals included in this study varied from 40 in 2012 to 78 in 2022 (Table S1). A total of 124 antibiotics were included, consisting of 24 antibiotic classes (Table S2). In 2022, the total inpatient antibiotic use in all study hospitals in Xinjiang was 27.6 DDDs/100 patient days. Antibiotic use in secondary hospitals was slightly higher than tertiary hospitals (28.5 vs. 27.2 DDDs/100 patient days). Between 2012 and 2022, the trend of inpatient antibiotic use for all hospitals showed a statistically significant decreasing trend (AAPC, −2.0%; 95% CI, −3.6% to −0.4%). While tertiary hospitals shared a similar trend (AAPC, −1.6%; 95% CI, −3.7% to 0.5%), a much fluctuated downward trend was observed for secondary hospitals (AAPC, −3.2%; 95% CI, −5.1% to −1.4%) (Figure 1). Yet, there was no significant difference in the trends of total antibiotic use for tertiary and secondary hospitals (Table S7).

In 2022, other β-lactam antibacterials (J01D) were the most used antibiotics, accounting for over half of the total antibiotics use (55.3%), followed by quinolone antibacterials (J01M) and β-lactam antibacterials and penicillins (J01C), accounting for 14.3% and 13.8% of the total antibiotic use, respectively (Table 1). Other β-lactam antibacterials (J01D) decreased significantly from 20.2 to 15.3 DDD/100 patient days (AAPC, −2.1%; 95% CI, −4.2% to 0.0%). A significant decreasing trend was also observed in penicillins (J01C) (AAPC, −3.6%; 95% CI, −5.5% to −1.6%), which was the second-most-used antibiotics classification from 2012 until 2017 but was surpassed by quinolone antibacterials (J01M) from 2018. Tetracyclines (J01A) were the only pharmacological sub-group that demonstrated a potential upward trend, even though this trend was not statistically significant (AAPC, 26.3%; 95% CI, −7.2% to 71.8%). In the pairwise comparison, both the test of coincidence and the test of parallelism showed significant differences in the antibiotic use of J01F and J01X for secondary and tertiary hospitals, indicating the trends for J01F and J01X were neither identical nor parallel between secondary and tertiary hospitals. (Table S7). The remaining parallel pairwise tests were not significant. Coincidence pairwise tests showed significant differences across hospital levels in all subgroups except J01C.

### 2.2. Antibiotic Usage by AWaRe Classification

**Figure 2:**
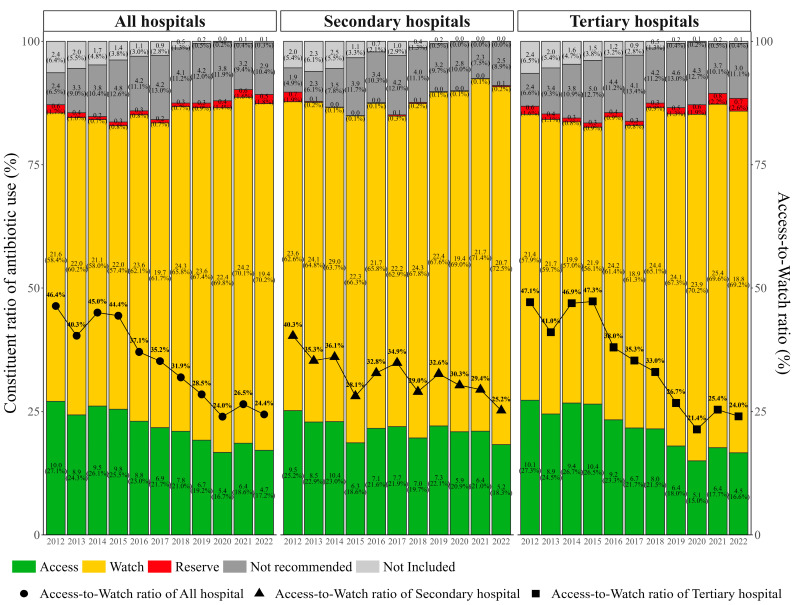
Constituent ratio of antibiotic use by hospital level in Xinjiang, 2012–2022.

When looking at antibiotic use under AWaRe classification, antibiotic use in the Access group decreased significantly from 10.0 to 4.7 DDD/100 patient days (AAPC, −6.7%; 95% CI, −8.8% to −4.5%) between 2012 and 2022. The proportion of antibiotic use in the Access group accounting for total antibiotic use dropped from 27.1% to 17.1% (Figure 2, Table S3). Although the antibiotic use in the Watch group, which accounted for the most of all antibiotic use, decreased slightly from 21.6 DDD/100 patient days in 2012 to 19.4 DDD/100 patient days in 2022, its overall trend over the whole study period was not downward (AAPC, 0.2%; 95% CI, −1.6% to 2.0%). The proportion of antibiotic use in the Watch group was observed to rise from 58.4% to 70.3% accordingly. As a result, the Access-to-Watch ratio decreased significantly from 46.4% in 2012 to 24.4% in 2022 (AAPC, −6.8%; 95% CI, −8.4% to −5.1%) (Figure 2). Moreover, the proportion of antibiotic use in the Not recommended group (14 antibiotics) accounted for more than 9.0% of the total use since 2013, even though its antibiotic use has moved downward in recent years. In addition, both tertiary and secondary hospitals showed a significant decrease in the Access-to-Watch ratio, with an AAPC of −7.9% (95% CI, −10.2% to −5.5%) and −3.1% (95% CI, −5.0% to −1.2%), respectively (Figure 2, Tables S6 and S7). The Access-to-Watch ratio remained above 30% for tertiary hospitals and higher than that for secondary hospitals until 2019, albeit the latter showed a much-fluctuated trend with a relatively more steady trend. In the pairwise comparison, a significant difference in the parallel test was found for antibiotic use in the “Not included” group between secondary and tertiary hospitals, while significant differences were found in the coincidence tests for the “Reserve” and “Not recommended” groups between the secondary and tertiary hospitals (Table S7).

### 2.3. DU90%

The number of antibiotics accounting for 90% of the total antibiotic use decreased from 34 antibiotics in 2012 to 18 antibiotics in 2022. Third-generation cephalosporins (J01DD, 28.6%), second-generation cephalosporins (J01DC, 20.6%), and fluoroquinolones (J01MA, 14.3%) were the top-three-used antibiotic classes during the study period. Antibiotic classes consisting of DU90% were similar for tertiary hospitals and secondary hospitals (Table S4).

### 2.4. Top-10 Antibiotics

**Figure 3:**
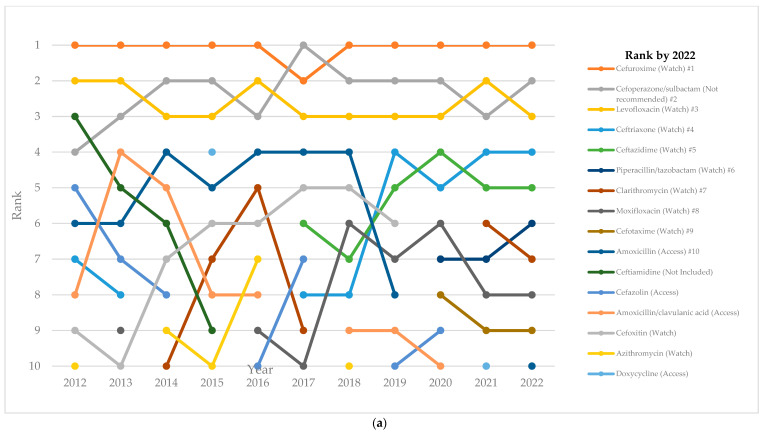

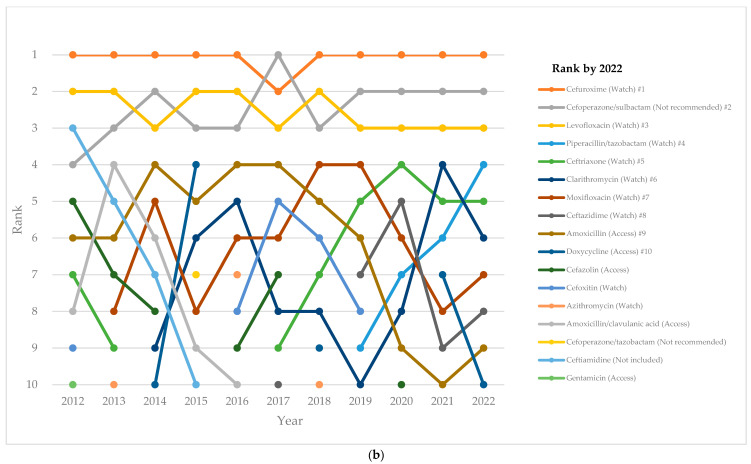

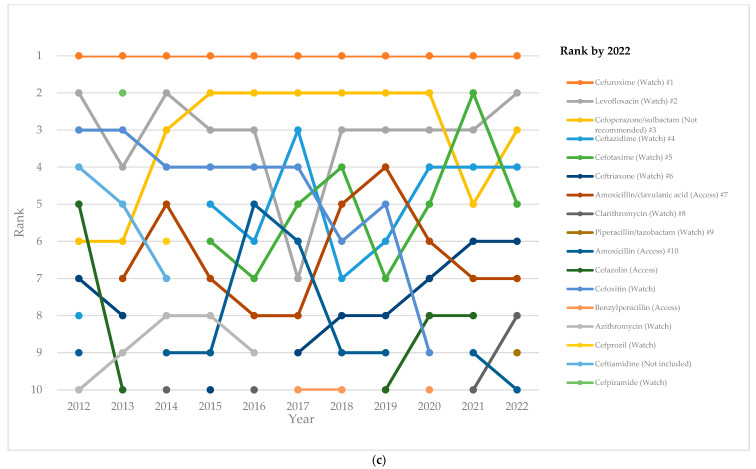
Ranking of the 10-most-used antibiotics among inpatients over time by hospital level: (**a**) all hospitals; (**b**) tertiary hospitals; and (**c**) secondary hospitals.

Figure 3 lists the top-10 antibiotics used in each study year, and it illustrates how the ranking of these antibiotics changed across from 2012 to 2022. Cefuroxime, cefoperazone/sulbactam, and levofloxacin were the most frequently used antibiotic (Figure 3a). The rankings of ceftriaxone and ceftazidime have been going up since 2018, while piperacillin/tazobactam has been ranked in the top 10 since 2020 for tertiary hospitals (Figure 3b) and from 2022 for secondary hospitals (Figure 3c). In 2022, 8 out of the top 10 antibiotics were in the Watch group. The composition of the top-10 antibiotics has been broadly similar in tertiary and secondary hospitals (Table S5).

## 3. Discussion

This study provides the most-recent estimates on systemic antibiotic use in the Northwest region of China with the longest time span up to date, allowing us to observe the secular trend of antibiotic use and quantifying the changes in antibiotic patterns over a 11-year period using the DDD metrics and AWaRe classification.

According to the national antibiotic stewardship requirement in 2015, the antibiotic use in secondary and tertiary hospitals in the public sector shall not exceed 40 DDDs/100 patient days [21]. This study found the total inpatient antibiotic use in Xinjiang between 2012–2022 to be lower than 40 DDDs/100 patient days, which is also lower than the neighboring provinces and coastal regions in China [15,19]. Xinjiang also had a lower antibiotic usage compared to some neighboring countries [22] and even some developed countries [23,24,25].

Moreover, the downward trend of inpatient antibiotic use in Xinjiang during the study period is in line with the national trend [15]. On the one hand, this suggests that the long-term improvement is due to a series of regulatory measures that was launched from 2011 with a 3-year national-level regulatory campaign that targeted public hospitals for the purpose of curbing antibiotic use [26]. On the other hand, although the economic development level of Xinjiang has been relatively lower compared with coastal regions, part of the reason for its lesser antibiotic use might lie in the small population in Xinjiang and the reduced severe disease spectrum, where patients with more-severe diseases would seek treatment in hospitals from more-developed regions. Nonetheless, Xinjiang suffered several surges of COVID-19 in the pandemic during 2020 and 2022, which led to a relatively longer period of lockdown compared to other provinces. This could also be a reason for the diminished number of hospitalization and, consequently, the decline in medical service utilization and the lesser antibiotic use. Despite these potential reasons, the sensitivity analysis results we performed on the data excluding 2020–2022 showed a decreasing trend for total antibiotic use from 2012 to 2019, albeit with no statistical significance, which supports the main finding in our base-case analysis.

Our findings indicate that the proportion of Access antibiotics continuously decreased to less than 20% between 2012 and 2022. It is concerning that this trend is moving further away from the goal set by WHO, where the Access group antibiotics should account for no less than 60% of total antibiotic use [27]. The low Access group antibiotic use in Xinjiang aligns with the national-level data, as well as other Asian countries [15,28]. One of the possible reasons for the low Access group antibiotic use might be driven by the guidance of the antibiotic restriction list, a critical regulation tool that was implemented at the provincial level from 2012 to promote rational antibiotic use. The antibiotics in the list were divided into three groups: non-restricted, restricted, and highly restricted. These groups are based on the prevalence of resistance, drug safety and efficacy, and its economic burden [17]. Hospitals are required to provide annual training on clinical knowledge and the standardized management of antibiotic use for physicians and pharmacists, and physicians are granted different levels of prescriptive authority for these antibiotics based on their specialty, job title, and clinical experience. A previous study showed that there is a large number of antibiotics classified as non-restricted in the Chinese restriction list but are also in the Watch group in the WHO AWaRe classification, such as levofloxacin, clarithromycin, azithromycin (oral form), and cefuroxime, the usage of which were among the top-10-most-used antibiotics in 2022 [29].

In our study, third-generation cephalosporins, second-generation cephalosporins, and fluoroquinolones remained the top-three antibiotic classes throughout the whole study period. The high usage of cephalosporins was in agreement with other regions of China and the national data [15,19]. The high usage of cephalosporins can be attributed not only to the inpatient setting, but also to the recommendation by the Chinese national guidelines for most perioperative prophylaxis indications [30]. This may partly be because penicillins require time-consuming skin allergy testing before administration, which contributes to the preference in using cephalosporins. In addition, our study also found that the usage of fixed-dose combination antibiotics, most of which were in the “Not recommended” group, takes a concerning proportion of the total antibiotic use, though the “Not recommended” antibiotics have started to move downward in recent years. For instance, cefoperazone/sulbactam, although used in China for decades but listed in the not recommended group, has been one of the top-three antibiotics used in Xinjiang since 2013. This highlights the importance of an evidence-based regulation of antibiotics. Further evidence in terms of the effectiveness and safety of these antibiotics are needed.

The disparities found in this study between tertiary and second hospitals on antibiotic usage, especially the significant differences found in the coincidence comparison in most ATC-3 subgroups, the “Reserve” group, and the “Not recommended” group, reflect structural differences in the antibiotic selection across hospital levels. China’s hospitals are basically classified according to the level of care. Tertiary hospitals are at the highest level, which provide specialist healthcare services, and the patients usually have more severe disease or present with more complicated conditions. Thus, the comparison findings stress the need for strengthening knowledge on the clinical application of antibiotics to meet different healthcare demands driven by various demographic and epidemiological patterns.

The major strengths of this study include the use of regional CAS data with broad coverage over a long period. The analysis included the latest available year of regional antibiotic use data. The use of Joinpoint regression for time-trend analysis allowed us to better interpret the longitudinal data. With its reliability and flexibility in dealing with large datasets, which has been demonstrated in previous studies [31,32], Joinpoint regression and the modified BIC method generates robust temporal-trend analysis results. Therefore, with regional representative data covering the largest sample size for Xinjiang public hospitals by far (to our knowledge), this comprehensive analysis of inpatient antibiotic usage in Xinjiang fills the region-specific evidence gaps. The valuable insights revealed in this study could serve as an instrumental monitoring tool to guide regional strategies more accurately in containing the impact of AMR. Furthermore, by following the methodology recommended by the WHO surveillance system and using indicators comparable over time and system, these temporal-trend analysis results provide a quantitative measure of the magnitude of change, and hence a means to reflect the efforts of the regional surveillance network, which allows for a progress comparison across regions.

This study has several limitations. First, the participation of the sample hospitals in CAS was voluntary-based, which may affect the representativeness due to selection bias. However, according to the China Health Statistic Yearbook, our sample hospitals accounted for 23.9% and 61.8% of the total number of secondary and tertiary public hospitals in Xinjiang [33]. In addition, the exclusion of ten hospitals from the original sample size may bring some bias, but the impact could be very limited. Nine of the ten hospitals were excluded because they did not report any inpatient antibiotic use data to the system at all, and the one hospital that was excluded due to key information missing is a psychiatric hospital with limited inpatients. According to the 2022 data, the only study year the given hospital reported inpatient-day numbers was when its annual inpatient days accounted for approximately 0.5% of the total inpatient days of all hospitals included in the analysis. Second, the lack of route of administration data in drug information makes it difficult to calculate the number of DDDs of antibiotics, including ornidazole, tinidazole, streptomycin, vancomycin, neomycin, and colistin, as well as the AWaRe classification of fosfomycin and minocycline, for which we classified into the reserve group regardless of route of administration. This would overestimate the use of these antibiotics. Third, the CAS database does not cover antibiotic use in primary health care institutions. However, this study is still of great value since we limited our analysis in the scope of inpatients, where the healthcare services were mainly provided by secondary and tertiary hospitals. Fourth, CAS only collect aggregated data for analysis, meaning no patient-level data of antibiotic use was available for further assessing the appropriateness of antibiotic use at the individual level.

## 4. Materials and Methods

### 4.1. Study Design

This was a cross-sectional analysis of an ecological study with a time-trend analysis on the inpatient antibiotic use in public hospitals in Xinjiang Uygur Autonomous Region of China from 1 January 2012 to 31 December 2022.

### 4.2. Setting

Xinjiang Uygur Autonomous Region is located in the northwestern part of China. It spans an area of 1.66 million square kilometers, constituting roughly one sixth of the country’s total land area. It is one of the largest provincial-level divisions in China with the longest land border, sharing borders with eight countries from Mongolia, Russia, and Kazakhstan in the north, all the way to Pakistan and India in the south. As of 2022, the region is inhabited by an estimated multi-ethnic population of around 26 million people [33]. The per capita GDP level of the region is USD 10.2 thousand, ranking 18th among the mainland provinces nationwide [33].

### 4.3. Data Source

This study utilized data from the Xinjiang’s Center for Antibacterial Surveillance (CAS) database, which is part of the national CAS database and is, by far, the largest nationwide surveillance database collecting data on antibacterial use from the 31 provinces of mainland China (excluding the Hong Kong Special Administrative Region, the Macao Special Administrative Region, and Taiwan, China). Participating hospitals of CAS upload quarterly data using a website-based system. The details of CAS have been published elsewhere [15]. This study was based on the secondary aggregated data on antibiotic use at the hospital level. No data of any kind on the individual level were involved. As such, ethical approval was not required.

We first included all 102 hospitals, including 40 tertiary hospitals and 62 secondary hospitals, which reported antibiotic use to Xinjiang’s CAS database at least once during the study period from January 2012 until December 2022. Ten hospitals were excluded due to their incomplete data. Nine hospitals were excluded because no inpatient antibiotic use data were uploaded to the system, and one hospital was excluded due to a lack of key information, such as the number of inpatient days. Finally, a total of 92 hospitals, including 37 tertiary hospitals and 56 secondary hospitals, were included in this study.

### 4.4. Data Collection

This study covered all antibiotics for systemic use, as defined by WHO’s Anatomical Therapeutic Chemical (ATC) classification J01 [34]. Hospital information including hospital name, hospital level, and the number of inpatient days were extracted from the database. Information on each antibiotic included the following: (i) the generic name; (ii) the drug classification; and (iii) the usage volume (i.e., the number of doses administered per quarter). Antibiotic use was expressed as the number of defined daily doses (DDDs) per patient days at the level of active substance. The usage of antibiotic was aggregated annually at the level of active substances (ATC Level 5) for each participating hospital in the CAS system and then exported for analysis.

### 4.5. Data Analysis

The trends and patterns of total antibiotic use were analyzed, and these were further described by different ATC level subgroups. The usage percentage of antibiotics under ATC levels in any given year was calculated by dividing the usage volume for each classification or individual antibiotic, as appropriate, by the total usage volume of all antibiotics in that year. The WHO Access, Watch, Reserve (AWaRe) classification of antibiotics (2023 version) [35] was employed to classify antibiotics to reflect their availability and potential impact on antimicrobial resistance. Other than the four groups of AWaRe classification (Access, Watch, Reserve, and Not Recommended), we introduced the fifth group of “Not Included” to specify antibiotics that are not in WHO’s AWaRe classification but were used in clinical practices in Xinjiang’s hospitals. The indicator of Access-to-Watch ratio was calculated to capture the composition of antibiotic use [36], which serves as an instrument to gauge the rational use of antibiotics.

Joinpoint regression was used to quantify the time trends of antibiotic use from 2012 to 2022. The average annual percentage changes (AAPCs) [37] with 95% confidence intervals (CIs) for the antibiotic usage over the study period were calculated using the weighted Bayesian Information Criteria (WBIC) model with a parametric method [38,39]. Pairwise comparison was further performed to test the parallelism and coincidence of antibiotic use between tertiary and secondary hospitals [40]. The parallel comparison examines whether the slopes of the regression lines are significantly different between the groups, while the coincident comparison examines whether the regression lines are significantly different from each other in both the slope and intercept [41].

Then, the number of antibiotics constituting 90% of the total antibiotic use (Drug Utilization 90, DU90%), measured at the level of chemical subgroups (ATC Level 4), were also analyzed. To better identify the most-commonly used antibiotics over time, we also tracked the rankings of the top-10 antibiotics used for each year at the chemical substance level (ATC Level 5) during the study period. All analyses mentioned above were further stratified by hospital levels. Considering the potential impact of the COVID-19 pandemics, we also performed a sensitivity analysis with the exclusion of data from 2020, 2021, and 2022.

### 4.6. Statistical Analysis

Descriptive analyses of antibiotic use were conducted. A *p*-value less than 0.05 was considered statistically significant. The data were managed, analyzed, and visualized using Microsoft Excel 2018 and Stata/SE 15.1 (StataCorp LLC, College Station, TX, USA), and Joinpoint regression was performed with Joinpoint Regression Program version 5.2.0 (National Cancer Institute, Calverton, MD, USA) [42].

## 5. Conclusions

In conclusion, our study found that the temporal trend of inpatient antibiotic use at tertiary and secondary hospitals in the Xinjiang region decreased significantly between 2012 and 2022. This could probably reflect the positive effects of continuous AMS implementation during the past decade. However, the patterns of antibiotic use based on evidence need to be emphasized in terms of antibiotic selection. More effort is needed to expand surveillance data, in addition to aggregated data, to the patient level in order to provide opportunity to further optimize the appropriateness of antibiotic use.

## Figures and Tables

**Table 1:** Inpatient antibiotic use by antibacterial classification (ATC Level 3) and hospital level in Xinjiang.

ATC Codes (Level 3)	Rate of Antibiotic Use Defined as Daily Doses Per 100 Patient Days (% of Total Antibiotic Use)											AAPC, %	Lower 95% CI	Upper 95% CI	*p*-Value
	Year														
	2012	2013	2014	2015	2016	2017	2018	2019	2020	2021	2022				
All Hospitals	36.9 (100.0)	36.5 (100.0)	36.3 (100.0)	38.3 (100.0)	38.1 (100.0)	31.9 (100.0)	37 (100.0)	35 (100.0)	32.1 (100.0)	34.6 (100.0)	27.6 (100.0)	−2.0	−3.6	−0.4	0.018 *
J01A	0.1 (0.3)	0.1 (0.4)	1.1 (3.1)	2.3 (6.1)	0.8 (2.2)	0.6 (2.0)	1.0 (2.6)	0.7 (1.9)	0.6 (1.7)	1.6 (4.6)	0.9 (3.4)	26.3	−7.2	71.8	0.137
J01B	0.0 (0.0)	0.0 (0.0)		0.0 (0.0)	0.0 (0.0)				0.0 (0.0)			-	-	-	-
J01C	5.0 (13.5)	5.3 (14.6)	5.3 (14.6)	5.2 (13.5)	5.7 (15.1)	4.3 (13.6)	4.8 (13.0)	4.5 (12.7)	3.6 (11.1)	4.1 (12.0)	3.8 (13.7)	−3.6	−5.5	−1.6	0.003 **
J01D	20.2 (54.7)	19.9 (54.6)	19.1 (52.5)	20.2 (52.7)	20.0 (52.4)	18.7 (58.7)	20.6 (55.7)	20.4 (58.2)	19.7 (61.4)	19.4 (56.0)	15.3 (55.3)	−2.1	−4.2	0.0	0.046 *
J01E	0.1 (0.3)	0.1 (0.2)	0.3 (0.9)	0.2 (0.5)	0.2 (0.4)	0.1 (0.4)	0.3 (0.7)	0.2 (0.5)	0.1 (0.3)	0.1 (0.4)	0.1 (0.4)	−1.4	−10.2	8.2	0.735
J01F	3.6 (9.7)	3.3 (9.0)	3.5 (9.6)	3.4 (9.0)	4.1 (10.9)	2.9 (9.2)	3.0 (8.1)	2.6 (7.5)	2.0 (6.3)	3.2 (9.3)	2.2 (8.1)	−4.3	−7.5	−0.9	0.018 *
J01G	1.8 (5.0)	1.2 (3.2)	0.8 (2.2)	0.6 (1.6)	0.6 (1.5)	0.6 (1.8)	0.6 (1.5)	0.4 (1.0)	0.3 (1.0)	0.2 (0.7)	0.2 (0.6)	−18.3	−21.3	−15.1	<0.001 ***
J01M	4.2 (11.3)	4.7 (12.9)	4.4 (12.2)	4.8 (12.4)	5.1 (13.4)	3.2 (9.9)	5.4 (14.6)	5.1 (14.7)	4.6 (14.2)	4.6 (13.3)	4 (14.3)	0.0	−3.3	3.4	0.979
J01X	1.9 (5.2)	1.9 (5.2)	1.8 (4.9)	1.6 (4.2)	1.6 (4.1)	1.4 (4.3)	1.4 (3.7)	1.2 (3.4)	1.3 (4.0)	1.3 (3.7)	1.1 (4.0)	−5.4	−6.4	−4.3	<0.001 ***
Tertiary Hospitals	36.8 (100.0)	36.4 (100.0)	35 (100.0)	39.1 (100.0)	39.4 (100.0)	30.9 (100.0)	37.4 (100.0)	35.9 (100.0)	34.1 (100.0)	36.5 (100.0)	27.2 (100.0)	−1.6	−3.7	0.5	0.111
J01A	0.1 (0.4)	0.1 (0.4)	1.3 (3.7)	2.8 (7.0)	1.1 (2.7)	0.8 (2.7)	1.3 (3.5)	0.9 (2.6)	0.8 (2.4)	2.3 (6.2)	1.2 (4.3)	30.1	−3.0	74.4	0.079
J01B	0.0 (0.0)	0.0 (0.0)		0.0 (0.0)	0.0 (0.0)				0.0 (0.0)			-	-	-	-
J01C	5.0 (13.6)	5.3 (14.6)	5.1 (14.6)	5.2 (13.3)	5.9 (15.0)	4.0 (12.9)	4.7 (12.5)	4.2 (11.7)	3.5 (10.4)	4.1 (11.3)	3.7 (13.6)	−3.9	−6.1	−1.6	0.004 **
J01D	19.9 (54.0)	19.5 (53.5)	18.0 (51.4)	20.0 (51.2)	19.9 (50.4)	17.3 (56.0)	19.5 (52.0)	19.7 (55.1)	20.0 (58.7)	19.0 (52.1)	14.2 (52.3)	−2.4	−5.8	1.1	0.180
J01E	0.1 (0.2)	0.1 (0.3)	0.4 (1.0)	0.2 (0.6)	0.2 (0.5)	0.2 (0.5)	0.4 (1.0)	0.2 (0.6)	0.1 (0.4)	0.2 (0.6)	0.2 (0.6)	10.1	−8.9	33.1	0.321
J01F	3.6 (9.7)	3.2 (8.9)	3.2 (9.2)	3.5 (8.9)	4.4 (11.2)	3.2 (10.3)	3.3 (8.8)	2.9 (8.2)	2.3 (6.8)	3.9 (10.6)	2.4 (8.9)	−2.6	−6.2	1.1	0.145
J01G	1.9 (5.3)	1.3 (3.5)	0.8 (2.3)	0.6 (1.6)	0.7 (1.7)	0.6 (1.9)	0.6 (1.6)	0.4 (1.2)	0.4 (1.2)	0.3 (0.8)	0.2 (0.7)	−17.0	−20.5	−13.5	<0.001 ***
J01M	4.3 (11.6)	4.9 (13.5)	4.5 (12.8)	5.0 (12.8)	5.6 (14.3)	3.5 (11.2)	6.2 (16.6)	6.0 (16.8)	5.3 (15.6)	5.2 (14.2)	4.1 (15)	0.8	−3.1	4.9	0.650
J01X	1.9 (5.2)	1.9 (5.4)	1.7 (5.0)	1.7 (4.4)	1.7 (4.2)	1.4 (4.4)	1.5 (4.0)	1.3 (3.7)	1.5 (4.5)	1.5 (4.2)	1.3 (4.7)	−3.6	−5.2	−1.9	0.001 **
Secondary Hospitals	37.8 (100.0)	37.2 (100.0)	45.5 (100.0)	33.6 (100.0)	32.9 (100.0)	35.2 (100.0)	35.8 (100.0)	33.1 (100.0)	28.1 (100.0)	30.4 (100.0)	28.5 (100.0)	−3.2	−5.1	−1.4	0.004 **
J01A	0.0 (0.1)	0.0 (0.1)	0.0 (0.1)	0.0 (0.0)	0.0 (0.0)	0.1 (0.2)	0.0 (0.1)	0.1 (0.2)	0.0 (0.1)	0.0 (0.1)	0.4 (1.4)	17.6	−6.2	47.6	0.140
J01C	4.6 (12.3)	5.5 (14.9)	6.7 (14.7)	4.9 (14.7)	5.0 (15.2)	5.5 (15.6)	5.2 (14.4)	5.0 (15.2)	3.6 (13.0)	4.2 (13.8)	4.0 (14.1)	−1.9	−8.1	4.6	0.553
J01D	23.2 (61.6)	23.5 (63.1)	26.7 (58.6)	21.0 (62.5)	20.4 (61.9)	23.4 (66.5)	23.6 (65.9)	21.8 (65.8)	19.0 (67.8)	20.1 (66.3)	17.7 (62.0)	−2.5	−4.3	−0.7	0.013 *
J01E	0.5 (1.4)	0.0 (0.0)	0.0 (0.0)	0.0 (0.0)	0.0 (0.0)	0.0 (0.0)	0.0 (0.0)	0.0 (0.0)	0.0 (0.0)	0.0 (0.0)	0.0 (0.0)	−22.6	−33.4	−10.0	0.001 **
J01F	3.4 (9.0)	3.4 (9.3)	5.2 (11.3)	3.1 (9.2)	3.1 (9.4)	2.1 (6.1)	2.3 (6.3)	2.0 (6.0)	1.5 (5.2)	1.8 (5.9)	1.8 (6.5)	−9.2	−13.1	−5.2	0.001 **
J01G	0.9 (2.5)	0.6 (1.5)	0.8 (1.7)	0.3 (1.0)	0.2 (0.7)	0.5 (1.3)	0.4 (1.2)	0.2 (0.6)	0.1 (0.4)	0.2 (0.5)	0.1 (0.4)	−17.9	−24.2	−11.2	<0.001 ***
J01M	3.1 (8.2)	2.7 (7.4)	4.1 (9.0)	3.3 (9.8)	3.0 (9.2)	2.2 (6.3)	3.2 (8.9)	3.2 (9.7)	3.1 (10.9)	3.3 (11.0)	3.7 (13.0)	0.7	−2.8	4.3	0.670
J01X	1.9 (5.0)	1.4 (3.7)	2.1 (4.6)	0.9 (2.8)	1.2 (3.6)	1.4 (4.0)	1.1 (3.1)	0.8 (2.6)	0.8 (2.7)	0.7 (2.3)	0.7 (2.5)	−9.3	−13.2	−5.3	0.001 **

Note 1: A blank in the table means no use of antibiotics of a given classification was reported in a given year. Note 2: The value of 0.0 in the table does not indicate an actual value of zero but represents a number that has been rounded to one decimal place, with subsequent decimal places not visible due to rounding. AAPC: Average Annual Percent Change and CI: confidence interval. * *p* < 0.05, ** *p* < 0.01, and *** *p* < 0.001.

## Data Availability

The extracted data that support the findings of this study are available from Xinjiang’s Center for Antibacterial Surveillance (CAS) database, but restrictions apply to the availability of these data, which were used under license for the current study, and thus are not publicly available. Data are, however, available from the authors upon reasonable request and with the permission of CAS.

## Supplementary Materials

The following supporting information can be downloaded at: https://www.mdpi.com/article/10.3390/antibiotics13080732/s1, Table S1: Number of included hospitals by hospital level. Table S2: List of antibiotics used in Xinjiang during the study period based on World Health Organization’s Access, Watch, Reserve classification (2023 version). Table S3: Inpatient antibiotic use by AWaRe classification and hospital level in Xinjiang. Table S4: Most-frequently used antibacterial classification (ATC4) accounting for 90% of inpatient use by hospital level, Xinjiang, 2012–2022. Table S5: Top-ten antibiotics among inpatients by hospital level and year in Xinjiang. Table S6: Access-to-Watch ratio results by hospital level in Xinjiang, 2012–2022. Table S7: Comparison results between tertiary and secondary hospitals.
