# Supplementary material for: Measuring Temporal Trends and Patterns of Inpatient Antibiotic Use in Northwest China’s Hospitals: Data from the Center for Antibacterial Surveillance, 2012–2022
Source: Antibiotics (Basel). 2024 Aug 5;13(8):732. doi: 10.3390/antibiotics13080732 (PMC11350706; doi:10.3390/antibiotics13080732)
Supplement: Supplementary file 1 [file antibiotics-13-00732-s001.zip › antibiotics-3048904-SI.pdf]

Supplementary Materials

Table S1. Number of included hospitals by hospital level.

Hospital level	Number of hospitals (%)										
	**2012**	**2013**	**2014**	**2015**	**2016**	**2017**	**2018**	**2019**	**2020**	**2021**	**2022**
All hospitals	40	43	49	50	57	68	70	77	76	78	78
Secondary hospitals	11 (27.5)	14 (32.6)	18 (36.7)	19 (38.0)	25 (43.9)	35 (51.5)	36 (51.4)	43 (55.8)	42 (55.3)	44 (56.4)	44 (56.4)
Tertiary hospitals	29 (72.5)	29 (67.4)	31 (63.3)	31 (62.0)	32 (56.1)	33 (48.5)	34 (48.6)	34 (44.2)	34 (44.7)	34 (43.6)	34 (43.6)

Table S2. List of antibiotics used in Xinjiang during the study period based on World Health Organization's Access, Watch, Reserve classification (2023 version).

Access antibiotics		ATC code
Tetracyclines	Doxycycline	J01AA02
	Tetracycline	J01AA07
Amphenicols	Chloramphenicol	J01BA01
Penicillins with extended spectrum	Ampicillin	J01CA01
	Amoxicillin	J01CA04
Beta-lactamase sensitive penicillins	Benzylpenicillin	J01CE01
	Phenoxymethylpenicillin	J01CE02
	Benzathine benzylpenicillin	J01CE08
	Procaine benzylpenicillin	J01CE09
Beta-lactamase resistant penicillins	Cloxacillin	J01CF02
	Oxacillin	J01CF04
	Flucloxacillin	J01CF05
Beta-lactamase inhibitors	Sulbactam	J01CG01
Combinations of penicillins, incl. beta-lactamase inhibitors	Ampicillin and Sulbactam	J01CR01
	Amoxicillin and Clavulanic acid	J01CR02
First-generation cephalosporins	Cefalexin	J01DB01
	Cefaloridine	J01DB02
	Cefalotin	J01DB03
	Cefazolin	J01DB04
	Cefazolin and Sulbactam	Not available
	Cefadroxil	J01DB05
	Cefapirin	J01DB08
	Cefradine	J01DB09
	Ceftezole	J01DB12
Intermediate-acting sulfonamides	Sulfamethoxazole	J01EC01
Trimethoprim and derivatives	Sulfamethoxazole and trimethoprim	J01EE01
Lincosamides	Clindamycin	J01FF01
Other aminoglycosides	Gentamicin	J01GB03
	Amikacin	J01GB06
Imidazole derivatives	Metronidazole (IV)	J01XD01

	Tinidazole (IV)	J01XD02
	Ornidazole (IV)	J01XD03
	**Watch antibiotics**	**ATC code**
Penicillins with an extended spectrum	Carbenicillin	J01CA03
	Azlocillin	J01CA09
	Mezlocillin	J01CA10
	Piperacillin	J01CA12
	Sulbenicillin	J01CA16
Combinations of penicillins, incl. beta-lactamase inhibitors	Piperacillin and Tazobactam	J01CR05
Second-generation cephalosporins	Cefoxitin	J01DC01
	Cefuroxime	J01DC02
	Cefamandole	J01DC03
	Cefaclor	J01DC04
	Cefonicide	J01DC06
	Cefotiam	J01DC07
	Cefmetazole	J01DC09
	Cefprozil	J01DC10
	Cefminox	J01DC12
Third-generation cephalosporins	Cefotaxime	J01DD01
	Ceftazidime	J01DD02
	Ceftriaxone	J01DD04
	Cefmenoxime	J01DD05
	Latamoxef	J01DD06
	Ceftizoxime	J01DD07
	Cefixime	J01DD08
	Cefodizime	J01DD09
	Cefetamet-pivoxil	J01DD10
	Cefpiramide	J01DD11
	Cefoperazone	J01DD12
	Cefpodoxime-proxetil	J01DD13
	Cefdinir	J01DD15
Fourth-generation cephalosporins	Cefepime	J01DE01
	Cefpirome	J01DE02
Carbapenems	Meropenem	J01DH02
	Ertapenem	J01DH03
	Biapenem	J01DH05
	Imipenem and cilastatin	J01DH51
	Panipenem	J01DH55
Macrolides	Erythromycin	J01FA01
	Midecamycin	J01FA03
	Roxithromycin	J01FA06
	Josamycin	J01FA07
	Clarithromycin	J01FA09
	Azithromycin	J01FA10
	Miocamycin	J01FA11
	Dirithromycin	J01FA13

Lincosamides	Lincomycin	J01FF02
Streptomycins	Streptomycin	J01GA01
Other aminoglycosides	Tobramycin	J01GB01
	Neomycin	J01GB05
	Netilmicin	J01GB07
Fluoroquinolones	Ofloxacin	J01MA01
	Ciprofloxacin	J01MA02
	Enoxacin	J01MA04
	Norfloxacin	J01MA06
	Lomefloxacin	J01MA07
	Fleroxacin	J01MA08
	Levofloxacin	J01MA12
	Moxifloxacin	J01MA14
	Gatifloxacin	J01MA16
	Pazufloxacin	J01MA18
Glycopeptides	Vancomycin (IV)	J01XA01
	Teicoplanin	J01XA02
Steroid antibacterials	Fusidic acid	J01XC01
	**Reserve antibiotics**	**ATC code**
Tetracyclines	Minocycline (IV)	J01AA08
	Tigecycline	J01AA12
Monobactams	Aztreonam	J01DF01
Polymyxins	Colistin	J01XB01
	Polymyxin B	J01XB02
Other antibacterials	Fosfomycin (IV)	J01XX01
	Linezolid	J01XX08
	**Not included antibiotics**	**ATC code**
Combinations of penicillins, incl. beta-lactamase inhibitors	Ticarcillin and Clavulanic acid	J01CR03
	Ampicillin and Probenecid	J01CR55
First-generation cephalosporins	Cephalexin and Trimethoprim	J01DB50
	Ceftiamidine	J01DB52
Third-generation cephalosporins	Cefpimizole	Not available
Fourth-generation cephalosporins	Ceftioli	J01DE30
Macrolides	Cyclic ester erythromycin	J01FA30
	Acetylspiramycin	J01FA50
	Etoerythromycin	J01FA51
	Erythromycin succinate	J01FA53
Streptogramins	Guitaromycin	J01FG50
Other aminoglycosides	Etimicin	J01GB51
Glycopeptide antibacterials	Norvancomycin	J01XA50
Other antibacterials	Fosfomycin tromethamine	J01XX74
	**Antibiotics not recommended**	**ATC code**
Combinations of penicillins, incl. beta-lactamase inhibitors	Amoxicillin and Sulbactam	J01CR51
	Ampicillin and Chloracillin	J01CR52
	Piperacillin and Sulbactam	J01CR54
	Amoxicillin and Dicloxacillin	J01CR56

	Amoxicillin and Sulbactam	J01CR57
	Meloxicillin and Sulbactam	J01CR58
Third-generation cephalosporins	Cefoperazone and Tazobactam	J01DD62
	Cefotaxime and Sulbactam	J01DD63
	Ceftriaxone and Sulbactam	J01DD66
	Cefoperazone and Sulbactam	J01DD68

Table S3. Inpatient antibiotic use by AWaRe classification and hospital level in Xinjiang.

AWaRe classification	Rate of antibiotic use by inpatients defined as daily doses per 100 patient days (% of total antibiotic use)											APC, %	Lower 95% CI	Upper 95% CI	P-value
	Year														
	2012	2013	2014	2015	2016	2017	2018	2019	2020	2021	2022				
**All hospitals**	**36.9(100.0)**	**36.5(100.0)**	**36.3(100.0)**	**38.3(100.0)**	**38.1(100.0)**	**31.9(100.0)**	**37.0(100.0)**	**35.0(100.0)**	**32.1(100.0)**	**34.6(100.0)**	**27.6(100.0)**	**-2.0**	**-3.6**	**-0.4**	**0.018***
Access	10.0(27.1)	8.9(24.3)	9.5(26.1)	9.8(25.5)	8.8(23.0)	6.9(21.8)	7.8(21.0)	6.7(19.2)	5.4(16.7)	6.4(18.6)	4.7(17.1)	-6.7	-8.8	-4.5	<0.001***
Watch	21.6(58.4)	22.0(60.2)	21.1(58.0)	22.0(57.4)	23.7(62.1)	19.7(61.7)	24.3(65.8)	23.6(67.4)	22.4(69.8)	24.2(70.1)	19.4(70.2)	0.2	-1.6	2.0	0.815
Reserve	0.6(1.7)	0.4(1.0)	0.2(0.7)	0.3(0.8)	0.3(0.8)	0.2(0.7)	0.3(0.7)	0.3(0.9)	0.4(1.4)	0.6(1.6)	0.5(1.9)	-2.0	-13.2	10.7	0.748
Not recommended	2.4(6.5)	3.3(9.0)	3.8(10.4)	4.8(12.6)	4.2(11.1)	4.2(13.0)	4.1(11.2)	4.2(12)	3.8(11.9)	3.2(9.4)	2.9(10.4)	2.4	-1.5	6.4	0.227
Not Included	2.4(6.4)	2.0(5.5)	1.7(4.8)	1.4(3.8)	1.1(3.0)	0.9(2.8)	0.5(1.3)	0.2(0.5)	0.0(0.1)	0.1(0.4)	0.1(0.3)	-32.7	-40.1	-24.4	<0.001***
**Tertiary hospitals**	**36.8(100.0)**	**36.4(100.0)**	**35.0(100.0)**	**39.1(100.0)**	**39.4(100.0)**	**30.9(100.0)**	**37.4(100.0)**	**35.9(100.0)**	**34.1(100.0)**	**36.5(100.0)**	**27.2(100.0)**	**-1.6**	**-3.7**	**0.5**	**0.111**
Access	10.1(27.3)	8.9(24.5)	9.4(26.7)	10.4(26.5)	9.2(23.3)	6.7(21.7)	8.0(21.5)	6.5(18.0)	5.1(15.0)	6.4(17.7)	4.5(16.6)	-7.2	-9.9	-4.4	<0.001***
Watch	21.4(57.9)	21.7(59.7)	20.0(57.0)	21.9(56.1)	24.2(61.4)	18.9(61.3)	24.4(65.1)	24.1(67.3)	23.9(70.2)	25.4(69.6)	18.8(69.2)	0.7	-1.7	3.1	0.539
Reserve	0.6(1.6)	0.4(1.1)	0.3(0.8)	0.3(0.9)	0.4(0.9)	0.3(0.8)	0.3(0.9)	0.5(1.3)	0.7(1.9)	0.8(2.2)	0.7(2.6)	6.2	-3.4	16.6	0.213
Not recommended	2.4(6.6)	3.4(9.3)	3.8(10.9)	5.0(12.7)	4.4(11.2)	4.1(13.4)	4.2(11.2)	4.7(13.0)	4.3(12.7)	3.7(10.1)	3.0(11.1)	3.2	-2.1	8.8	0.246
Not Included	2.4(6.5)	2.0(5.4)	1.6(4.6)	1.5(3.8)	1.2(3.2)	0.9(2.8)	0.5(1.3)	0.2(0.5)	0.1(0.2)	0.2(0.5)	0.1(0.4)	-30.3	-37.6	-22.1	<0.001***

**Secondary hospitals**	**37.8 (100.0)**	**37.2 (100.0)**	**45.5 (100.0)**	**33.6 (100.0)**	**32.9 (100.0)**	**35.2 (100.0)**	**35.8 (100.0)**	**33.1 (100.0)**	**28.1 (100.0)**	**30.4 (100.0)**	**28.5 (100.0)**	**-3.2**	**-5.1**	**-1.4**	**0.004 ****
Access	9.5(25.2)	8.5(22.9)	10.5 (23.0)	6.3(18.6)	7.1(21.6)	7.7(22.0)	7.0(19.7)	7.3(22.1)	5.9(20.9)	6.4(21.0)	5.2(18.3)	-5.0	-7.5	-2.3	0.002**
Watch	23.6 (62.6)	24.1 (64.8)	29.0 (63.7)	22.3 (66.3)	21.7 (65.8)	22.2 (62.9)	24.3 (67.7)	22.4 (67.6)	19.4 (68.9)	21.7 (71.4)	20.7 (72.5)	-2.0	-3.7	-0.1	0.038*
Reserve	0.7(1.9)	0.1(0.2)	0.0(0.1)	0.0(0.0)	0.0(0.1)	0.1(0.3)	0.1(0.2)	0.0(0.1)	0.0(0.1)	0.0(0.1)	0.1(0.3)	-20.8	-47.7	19.9	0.270
Not recommended	1.9(4.9)	2.3(6.1)	3.6(7.8)	3.9(11.7)	3.4(10.3)	4.2(12.0)	4.0(11.1)	3.2(9.7)	2.8(10.0)	2.3(7.5)	2.5(8.9)	2.9	-3.7	10.0	0.395
Not Included	2.0(5.4)	2.3(6.1)	2.5(5.5)	1.1(3.3)	0.7(2.1)	1.0(2.9)	0.5(1.3)	0.2(0.5)	0.0(0.1)	0.0(0.0)	0.0(0.0)	-45.1	-57.6	-28.8	< 0.001***

Note: The value of 0.0 in the table does not indicate an actual value of zero but represents a number that has been rounded to one decimal place, with subsequent decimal places not visible due to rounding.
AWaRe: Access, Watch, Reserve; AAPC: Average Annual Percent Change; and CI: confidence interval.
* p<0.05, ** p<0.01, and *** p<0.001

Table S4. Most-frequently used antibacterial classification (ATC4) accounting for 90% of inpatient use by hospital level, Xinjiang, 2012-2022.

**ATC codes (Level 4)**	**Rank and Rate of Antibiotic Use defined as daily doses per 100 patient days (% of total antibiotic use), by year**																					
	**Year**																					
	**2012**		**2013**		**2014**		**2015**		**2016**		**2017**		**2018**		**2019**		**2020**		**2021**		**2022**	
	Rank	Rate (%)	Rank	Rate (%)	Rank	Rate (%)	Rank	Rate (%)	Rank	Rate (%)	Rank	Rate (%)	Rank	Rate (%)	Rank	Rate (%)	Rank	Rate (%)	Rank	Rate (%)	Rank	Rate (%)
**All hospitals**																						
J01DD	2	6.2(16.8)	2	6.7(18.3)	2	6.9(19)	1	8.3(21.8)	2	7.8(20.6)	1	8.7(27.1)	2	8.7(23.5)	1	9.0(25.9)	1	9.6(30.0)	1	9.3(26.8)	1	7.9(28.6)
J01DC	1	8.1(21.9)	1	8.2(22.5)	1	7.6(21)	2	7.6(19.9)	1	8.2(21.4)	2	6.8(21.3)	1	9.3(25.2)	2	8.9(25.5)	2	7.9(24.6)	2	7.7(22.2)	2	5.7(20.6)
J01MA	4	4.2(11.3)	3	4.7(12.9)	3	4.4(12.2)	3	4.8(12.4)	3	5.1(13.4)	3	3.2(9.9)	3	5.4(14.6)	3	5.1(14.7)	3	4.6(14.2)	3	4.6(13.3)	3	4.0(14.3)
J01CR	6	2.3(6.2)	5	2.9(7.8)	6	2.6(7.2)	8	2.3(6.1)	6	2.4(6.4)	7	1.7(5.3)	5	2.3(6.3)	4	2.4(6.9)	4	2.3(7.1)	5	2.7(7.7)	4	2.4(8.9)
J01FA	5	3.1(8.3)	6	2.8(7.7)	5	3.1(8.5)	4	3.1(8)	4	3.8(9.9)	4	2.7(8.5)	4	2.7(7.2)	5	2.3(6.6)	5	1.7(5.4)	4	2.9(8.5)	5	2.1(7.4)
J01CA	7	2.0(5.4)	7	1.9(5.2)	7	2.2(6.1)	6	2.4(6.4)	5	2.8(7.5)	5	2.1(6.7)	6	1.9(5)	6	1.5(4.3)	7	0.9(2.9)	8	1.1(3.1)	6	1.0(3.8)

J01AA	N/A	N/A	N/A	N/A	9	1.1(3.1)	7	2.3(6.1)	N/A	N/A	N/A	N/A	9	1.0(2.6)	N/A	N/A	N/A	N/A	6	1.6(4.6)	7	0.9(3.4)
J01DH	N/A	N/A	N/A	N/A	N/A	N/A	N/A	N/A	9	1.0(2.5)	9	0.9(2.7)	N/A	N/A	8	0.9(2.5)	8	0.9(2.8)	N/A	N/A	8	0.9(3.1)
J01DB	3	4.4(12.0)	4	3.7(10.1)	4	3.3(9.1)	5	2.7(7.1)	7	2.4(6.4)	6	2.1(6.5)	7	1.5(4.1)	7	1.5(4.2)	6	1.2(3.9)	7	1.3(3.9)	N/A	N/A
J01XD	9	1.6(4.5)	8	1.5(4.2)	8	1.5(4.1)	9	1.3(3.3)	8	1.2(3.1)	8	1.0(3.3)	8	1.0(2.7)	N/A	N/A	N/A	N/A	N/A	N/A	N/A	N/A
J01GB	8	1.7(4.7)	9	1.1(3.0)	N/A	N/A	N/A	N/A	N/A	N/A	N/A	N/A	N/A	N/A	N/A	N/A	N/A	N/A	N/A	N/A	N/A	N/A
**Tertiary hospitals**																						
J01DD	2	6.2(16.8)	2	6.5(17.7)	2	6.7(19.1)	1	8.3(21.2)	1	7.8(19.9)	1	7.9(25.6)	2	8.2(22.0)	1	9.0(25.1)	1	9.8(28.7)	1	9.0(24.6)	1	7.3(26.7)
J01DC	1	7.7(21.0)	1	7.9(21.7)	1	6.8(19.3)	2	7.2(18.4)	2	7.6(19.2)	2	5.8(18.9)	1	8.3(22.1)	2	8.1(22.6)	2	7.8(23)	2	7.5(20.7)	2	5.1(18.7)
J01MA	4	4.3(11.6)	3	4.9(13.5)	3	4.5(12.8)	3	5.0(12.8)	3	5.6(14.3)	3	3.5(11.2)	3	6.2(16.6)	3	6.0(16.8)	3	5.3(15.6)	3	5.2(14.2)	3	4.1(15)
J01CR	6	2.4(6.5)	5	2.9(7.9)	6	2.5(7.1)	8	2.3(6.0)	6	2.6(6.5)	7	1.6(5.3)	5	2.2(5.9)	5	2.3(6.4)	4	2.2(6.6)	5	2.7(7.4)	4	2.4(8.8)
J01FA	5	3.1(8.5)	6	2.9(7.9)	5	2.9(8.3)	4	3.2(8.1)	4	4.1(10.4)	4	3.0(9.7)	4	3.0(8.0)	4	2.7(7.4)	5	2.1(6.2)	4	3.6(9.9)	5	2.2(8.2)
J01AA	N/A	N/A	N/A	N/A	9	1.3(3.7)	6	2.8(7.0)	N/A	N/A	N/A	N/A	8	1.3(3.5)	9	0.9(2.6)	N/A	N/A	6	2.3(6.2)	6	1.2(4.3)
J01CA	7	2.0(5.4)	7	1.9(5.1)	7	2.2(6.1)	7	2.5(6.4)	5	3.0(7.7)	6	2.0(6.4)	6	2.0(5.4)	6	1.5(4.3)	8	1.0(3.0)	8	1.1(3.1)	7	1.0(3.9)
J01DH	N/A	N/A	N/A	N/A	10	0.8(2.4)	N/A	N/A	9	1.2(3.0)	8	1.1(3.4)	9	1.1(2.9)	8	1.2(3.3)	6	1.3(3.7)	7	1.4(3.8)	8	1.0(3.8)
J01DB	3	4.5(12.2)	4	3.7(10.1)	4	3.2(9.1)	5	2.8(7.2)	7	2.6(6.5)	5	2.0(6.5)	7	1.6(4.3)	7	1.4(3.9)	7	1.1(3.2)	9	1.1(3.0)	9	0.8(2.9)
J01XD	9	1.6(4.4)	8	1.6(4.3)	8	1.4(4)	9	1.3(3.4)	8	1.2(3.1)	9	1.0(3.1)	N/A	N/A	N/A	N/A	N/A	N/A	N/A	N/A	N/A	N/A
J01GB	8	1.8(4.9)	9	1.2(3.2)	N/A	N/A	N/A	N/A	N/A	N/A	N/A	N/A	N/A	N/A	N/A	N/A	N/A	N/A	N/A	N/A	N/A	N/A
**Secondary hospitals**																						
J01DD	2	6.4(17)	2	8.6(23.2)	2	8.3(18.3)	2	8.5(25.3)	2	7.8(23.8)	1	11.1(31.4)	2	9.9(27.7)	2	9.2(27.8)	1	9.3(33.1)	1	9.9(32.7)	1	9.3(32.7)
J01DC	1	11.5(30.5)	1	10.7(28.8)	1	13.8(30.4)	1	10.1(29.9)	1	10.5(31.8)	2	9.9(28.1)	1	12.2(34.1)	1	10.8(32.6)	2	8.0(28.6)	2	8.0(26.5)	2	7.0(24.7)

J01MA	4	3.1(8.2)	4	2.7(7.4)	5	4.1(9.0)	3	3.3(9.8)	3	3.0(9.2)	5	2.2(6.3)	3	3.2(8.9)	3	3.2(9.7)	3	3.1(10.9)	3	3.3(11)	3	3.7(13)
J01CR	8	1.4(3.8)	5	2.7(7.3)	6	3.3(7.4)	5	2.2(6.7)	6	2.0(6.1)	6	1.9(5.3)	4	2.6(7.2)	4	2.7(8.1)	4	2.3(8.2)	4	2.6(8.6)	4	2.6(9.0)
J01FA	5	2.4(6.5)	6	2.4(6.6)	3	4.3(9.4)	4	2.5(7.3)	4	2.6(7.8)	7	1.8(5.2)	5	1.8(5.0)	6	1.5(4.5)	6	1.0(3.5)	6	1.5(4.8)	5	1.6(5.7)
J01CA	6	2.2(5.7)	7	2.1(5.8)	7	2.6(5.7)	7	1.9(5.8)	5	2.1(6.5)	3	2.6(7.4)	6	1.4(4.0)	7	1.4(4.3)	N/A	N/A	7	0.9(3.0)	6	1.0(3.5)
J01DB	3	4(10.5)	3	3.9(10.4)	4	4.3(9.4)	6	2.2(6.6)	7	1.9(5.8)	4	2.2(6.3)	7	1.3(3.6)	5	1.6(4.9)	5	1.5(5.5)	5	1.9(6.2)	7	0.9(3.1)
J01XD	7	1.9(4.9)	8	1.4(3.7)	8	2(4.5)	N/A	N/A	N/A	N/A	N/A	N/A	N/A	N/A	N/A	N/A	7	0.7(2.4)	N/A	N/A	N/A	N/A
J01FF	9	1.0(2.5)	N/A	N/A	N/A	N/A	N/A	N/A	N/A	N/A	N/A	N/A	N/A	N/A	N/A	N/A	N/A	N/A	N/A	N/A	N/A	N/A
J01GB	10	0.9(2.4)	N/A	N/A	N/A	N/A	N/A	N/A	N/A	N/A	N/A	N/A	N/A	N/A	N/A	N/A	N/A	N/A	N/A	N/A	N/A	N/A

N/A: not applicable.

Table S5. Top-ten antibiotics among inpatients by hospital level and year in Xinjiang.

Antibiotics	AWaRe classification	Rank and Rate of Antibiotic use defined as daily doses per 100 patient days (% of total antibiotic use), by year																					
		Year																					
		2012		2013		2014		2015		2016		2017		2018		2019		2020		2021		2022	
		Rank	Rate (%)	Rank	Rate (%)	Rank	Rate (%)	Rank	Rate (%)	Rank	Rate (%)	Rank	Rate (%)	Rank	Rate (%)	Rank	Rate (%)	Rank	Rate (%)	Rank	Rate (%)	Rank	Rate (%)
All hospitals																							
Cefuroxime	Watch	1	4.2 (11.5)	1	5.2 (14.3)	1	4.5 (12.3)	1	4.6 (12)	1	5 (13.2)	2	3.4 (10.6)	1	6.1 (16.5)	1	6.0 (17.2)	1	6.1 (19.1)	1	6.5 (18.9)	1	5.4 (19.7)
Cefoperazone and Sulbactam	Not recommended	4	1.9 (5.2)	3	2.7 (7.3)	2	3.1 (8.7)	2	3.4 (8.9)	3	3.4 (8.8)	1	3.6 (11.2)	2	3.8 (10.1)	2	3.8 (10.8)	2	3.8 (11.8)	3	3.1 (9.0)	2	2.8 (10.3)
Levofloxacin	Watch	2	2.7 (7.4)	2	3.0 (8.4)	3	2.9 (8.1)	3	3.4 (8.8)	2	3.6 (9.5)	3	2.1 (6.6)	3	3.7 (10.1)	3	3.5 (10)	3	3.2 (10.1)	2	3.1 (9.0)	3	2.8 (10.1)
Ceftriaxone	Watch	7	1.5 (4.2)	8	1.3 (3.5)	N/A	N/A	N/A	N/A	N/A	N/A	8	1.1 (3.5)	8	1.4 (3.7)	4	1.8 (5)	5	1.9 (5.8)	4	2.0 (5.9)	4	1.7 (6.2)
Ceftazidime	Watch	N/A	N/A	N/A	N/A	N/A	N/A	N/A	N/A	N/A	N/A	6	1.4 (4.5)	7	1.4 (3.8)	5	1.6 (4.5)	4	2.0 (6.3)	5	1.9 (5.6)	5	1.6 (5.7)
Piperacillin and Tazobactam	Watch	N/A	N/A	N/A	N/A	N/A	N/A	N/A	N/A	N/A	N/A	N/A	N/A	N/A	N/A	N/A	N/A	7	1.2 (3.7)	7	1.6 (4.6)	6	1.5 (5.6)
Clarithromycin	Watch	N/A	N/A	N/A	N/A	10	1.2 (3.4)	7	1.4 (3.6)	5	1.6 (4.3)	9	1 (3.2)	N/A	N/A	N/A	N/A	N/A	N/A	6	1.9 (5.6)	7	1.4 (5.0)
Moxifloxacin	Watch	N/A	N/A	9	1.3 (3.5)	N/A	N/A	N/A	N/A	9	1.3 (3.4)	10	1 (3.1)	6	1.6 (4.2)	7	1.6 (4.4)	6	1.3 (4.0)	8	1.4 (4.1)	8	1.1 (4.0)
Cefotaxime	Watch	N/A	N/A	N/A	N/A	N/A	N/A	N/A	N/A	N/A	N/A	N/A	N/A	N/A	N/A	N/A	N/A	8	1.2 (3.7)	9	1.3 (3.9)	9	1.1 (3.9)
Amoxicillin	Access	6	1.6 (4.3)	6	1.5 (4.1)	4	1.9 (5.2)	5	2.1 (5.6)	4	2.6 (6.9)	4	1.8 (5.8)	4	1.7 (4.5)	8	1.3 (3.8)	N/A	N/A	N/A	N/A	10	1.0 (3.4)
Ceftiamidine	Not Included	3	2.0 (5.3)	5	1.7 (4.5)	6	1.4 (3.9)	9	1.2 (3.1)	N/A	N/A	N/A	N/A	N/A	N/A	N/A	N/A	N/A	N/A	N/A	N/A	N/A	N/A
Cefazolin	Access	5	1.8 (4.7)	7	1.4 (4)	8	1.3 (3.5)	N/A	N/A	10	1.2 (3.2)	7	1.1 (3.5)	N/A	N/A	10	1.1 (3)	9	1.1 (3.4)	N/A	N/A	N/A	N/A

Amoxicillin and Clavulanic acid	Access	8	1.5 (4)	4	1.8 (4.9)	5	1.5 (4.1)	8	1.2 (3.2)	8	1.3 (3.4)	N/A	N/A	9	1.3 (3.6)	9	1.3 (3.7)	10	1.1 (3.3)	N/A	N/A	N/A	N/A
Cefoxitin	Watch	9	1.4 (3.9)	10	1.3 (3.4)	7	1.4 (3.9)	6	1.4 (3.6)	6	1.5 (4.0)	5	1.8 (5.7)	5	1.6 (4.3)	6	1.6 (4.5)	N/A	N/A	N/A	N/A	N/A	N/A
Azithromycin	Watch	10	1.2 (3.2)	N/A	N/A	9	1.2 (3.4)	10	1.1 (3.0)	7	1.4 (3.7)	N/A	N/A	10	1.1 (3.1)	N/A	N/A	N/A	N/A	N/A	N/A	N/A	N/A
Doxycycline	Access	N/A	N/A	N/A	N/A	N/A	N/A	4	2.2 (5.8)	N/A	N/A	N/A	N/A	N/A	N/A	N/A	N/A	N/A	N/A	10	1.3 (3.7)	N/A	N/A
**Tertiary hospitals**																							
Cefuroxime	Watch	1	3.9 (10.5)	1	4.9 (13.6)	1	4.1 (11.6)	1	4.3 (10.9)	1	4.5 (11.4)	2	2.6 (8.4)	1	4.9 (13.1)	1	5.1 (14.3)	1	6.0 (17.5)	1	6.3 (17.3)	1	4.8 (17.7)
Cefoperazone and Sulbactam	Not recommended	4	1.9 (5.3)	3	2.7 (7.5)	2	3.1 (8.9)	3	3.4 (8.6)	3	3.4 (8.7)	1	3.5 (11.2)	3	3.8 (10.1)	2	4.1 (11.4)	2	4.3 (12.6)	2	3.5 (9.6)	2	3.0 (11.0)
Levofloxacin	Watch	2	2.7 (7.4)	2	3.1 (8.6)	3	2.9 (8.2)	2	3.5 (8.9)	2	3.9 (9.9)	3	2.2 (7)	2	4.1 (11)	3	3.9 (10.8)	3	3.6 (10.5)	3	3.4 (9.2)	3	2.8 (10.2)
Piperacillin and Tazobactam	Watch	N/A	N/A	N/A	N/A	N/A	N/A	N/A	N/A	N/A	N/A	N/A	N/A	N/A	N/A	9	1.2 (3.4)	7	1.5 (4.5)	6	2.0 (5.4)	4	1.8 (6.6)
Ceftriaxone	Watch	7	1.6 (4.2)	9	1.2 (3.4)	N/A	N/A	N/A	N/A	N/A	N/A	9	1.1 (3.5)	7	1.4 (3.9)	5	1.8 (4.9)	4	2.0 (5.8)	5	2.1 (5.7)	5	1.6 (6.0)
Clarithromycin	Watch	N/A	N/A	N/A	N/A	9	1.2 (3.5)	6	1.5 (3.7)	5	1.8 (4.6)	8	1.1 (3.6)	8	1.2 (3.3)	10	1.2 (3.2)	8	1.0 (3.0)	4	2.5 (6.8)	6	1.6 (5.7)
Moxifloxacin	Watch	N/A	N/A	8	1.4 (3.9)	5	1.3 (3.8)	8	1.2 (3.2)	6	1.5 (3.8)	6	1.2 (3.9)	4	2.0 (5.4)	4	2.1 (5.9)	6	1.7 (5.1)	8	1.8 (4.9)	7	1.3 (4.6)
Ceftazidime	Watch	N/A	N/A	N/A	N/A	N/A	N/A	N/A	N/A	N/A	N/A	10	1 (3.3)	N/A	N/A	7	1.4 (3.9)	5	1.9 (5.4)	9	1.7 (4.7)	8	1.2 (4.5)
Amoxicillin	Access	6	1.6 (4.4)	6	1.5 (4.2)	4	1.9 (5.5)	5	2.3 (5.9)	4	2.8 (7.2)	4	1.8 (5.9)	5	1.9 (5.1)	6	1.4 (4)	9	1.0 (3.0)	10	1.1 (3.0)	9	1.0 (3.6)
Doxycycline	Access	N/A	N/A	N/A	N/A	10	1.2 (3.3)	4	2.6 (6.7)	N/A	N/A	N/A	N/A	9	1.2 (3.3)	N/A	N/A	N/A	N/A	7	1.9 (5.1)	10	0.9 (3.2)
Cefazolin	Access	5	1.8 (4.8)	7	1.5 (4)	8	1.3 (3.7)	N/A	N/A	9	1.3 (3.3)	7	1.2 (3.9)	N/A	N/A	N/A	N/A	10	1.0 (2.9)	N/A	N/A	N/A	N/A

Cefoxitin	Watch	9	1.4 (3.7)	N/A	N/A	N/A	N/A	N/A	N/A	8	1.4 (3.5)	5	1.6 (5.2)	6	1.5 (3.9)	8	1.4 (3.8)	N/A	N/A	N/A	N/A	N/A	N/A
Azithromycin	Watch	N/A	N/A	10	1.2 (3.3)	N/A	N/A	N/A	N/A	7	1.4 (3.7)	N/A	N/A	10	1.2 (3.2)	N/A	N/A	N/A	N/A	N/A	N/A	N/A	N/A
Amoxicillin and Clavulanic acid	Access	8	1.5 (4.2)	4	1.8 (4.8)	6	1.3 (3.8)	9	1.2 (3.1)	10	1.3 (3.3)	N/A	N/A	N/A	N/A	N/A	N/A	N/A	N/A	N/A	N/A	N/A	N/A
Cefoperazone and Tazobactam	Not recommended	N/A	N/A	N/A	N/A	N/A	N/A	7	1.3 (3.3)	N/A	N/A	N/A	N/A	N/A	N/A	N/A	N/A	N/A	N/A	N/A	N/A	N/A	N/A
Ceftiamidine	Not Included	3	2.0 (5.4)	5	1.6 (4.4)	7	1.3 (3.8)	10	1.2 (3.1)	N/A	N/A	N/A	N/A	N/A	N/A	N/A	N/A	N/A	N/A	N/A	N/A	N/A	N/A
Gentamicin	Access	10	1.2 (3.3)	N/A	N/A	N/A	N/A	N/A	N/A	N/A	N/A	N/A	N/A	N/A	N/A	N/A	N/A	N/A	N/A	N/A	N/A	N/A	N/A
**Secondary hospitals**																							
Cefuroxime	Watch	1	7.7 (20.3)	1	7.6 (20.4)	1	7.3 (15.9)	1	6.3 (18.7)	1	7.2 (21.9)	1	5.9 (16.7)	1	9.4 (26.1)	1	8 (24.3)	1	6.5 (23.0)	1	7.1 (23.4)	1	6.9 (24.3)
Levofloxacin	Watch	2	2.7 (7.1)	4	2.4 (6.4)	2	3.4 (7.4)	3	2.8 (8.2)	3	2.5 (7.6)	7	1.9 (5.3)	3	2.8 (7.7)	3	2.7 (8.1)	3	2.6 (9.2)	3	2.6 (8.6)	2	2.8 (9.9)
Cefoperazone and Sulbactam	Not recommended	6	1.5 (4.1)	6	2.1 (5.6)	3	3.3 (7.2)	2	3.6 (10.8)	2	3.1 (9.5)	2	3.9 (11.1)	2	3.7 (10.4)	2	3.1 (9.4)	2	2.8 (10.0)	5	2.2 (7.3)	3	2.5 (8.8)
Ceftazidime	Watch	8	1.3 (3.6)	N/A	N/A	N/A	N/A	5	1.7 (5.1)	6	1.7 (5.2)	3	2.7 (7.7)	7	1.9 (5.4)	6	2 (6.2)	4	2.4 (8.6)	4	2.5 (8.3)	4	2.4 (8.3)
Cefotaxime	Watch	N/A	N/A	N/A	N/A	N/A	N/A	6	1.4 (4.1)	7	1.3 (4.0)	5	2.2 (6.3)	4	2.1 (6.0)	7	1.8 (5.4)	5	2.3 (8.3)	2	3.0 (9.7)	5	2.2 (7.6)
Ceftriaxone	Watch	7	1.4 (3.7)	8	1.7 (4.5)	N/A	N/A	10	1.1 (3.4)	N/A	N/A	9	1.1 (3.2)	8	1.2 (3.3)	8	1.8 (5.3)	7	1.6 (5.8)	6	1.9 (6.4)	6	1.9 (6.7)
Amoxicillin and Clavulanic acid	Access	N/A	N/A	7	1.9 (5.2)	5	2.5 (5.6)	7	1.3 (3.9)	8	1.3 (4.0)	8	1.3 (3.8)	5	2.0 (5.6)	4	2.2 (6.7)	6	1.9 (6.6)	7	1.9 (6.3)	7	1.6 (5.7)
Clarithromycin	Watch	N/A	N/A	N/A	N/A	10	1.4 (3.0)	N/A	N/A	10	1 (3.0)	N/A	N/A	N/A	N/A	N/A	N/A	N/A	N/A	10	0.7 (2.4)	8	1.0 (3.5)

Piperacillin and Tazobactam	Watch	N/A	N/A	N/A	N/A	N/A	N/A	N/A	N/A	N/A	N/A	N/A	N/A	N/A	N/A	N/A	N/A	N/A	N/A	N/A	N/A	9	0.9 (3.2)
Amoxicillin	Access	9	1.3 (3.5)	N/A	N/A	9	1.7 (3.6)	9	1.1 (3.4)	5	1.8 (5.4)	6	1.9 (5.3)	9	1.1 (3.0)	9	1.2 (3.5)	N/A	N/A	9	0.8 (2.6)	10	0.9 (3.0)
Cefazolin	Access	5	1.8 (4.7)	10	1.3 (3.4)	N/A	N/A	N/A	N/A	N/A	N/A	N/A	N/A	N/A	N/A	10	1.2 (3.5)	8	1.4 (4.9)	8	1.5 (4.9)	N/A	N/A
Cefoxitin	Watch	3	2.1 (5.5)	3	2.5 (6.7)	4	3.2 (7.0)	4	2.3 (6.9)	4	2.1 (6.5)	4	2.5 (7.1)	6	2.0 (5.6)	5	2.1 (6.2)	9	1.2 (4.3)	N/A	N/A	N/A	N/A
Benzylpenicillin	Access	N/A	N/A	N/A	N/A	N/A	N/A	N/A	N/A	N/A	N/A	10	1 (2.8)	10	1.0 (2.8)	N/A	N/A	10	0.6 (2.2)	N/A	N/A	N/A	N/A
Azithromycin	Watch	10	1.3 (3.4)	9	1.3 (3.6)	8	1.9 (4.1)	8	1.2 (3.7)	9	1.3 (4)	N/A	N/A	N/A	N/A	N/A	N/A	N/A	N/A	N/A	N/A	N/A	N/A
Cefprozil	Watch	N/A	0.3 (0.8)	N/A	N/A	6	2.2 (4.8)	N/A	N/A	N/A	N/A	N/A	N/A	N/A	N/A	N/A	N/A	N/A	N/A	N/A	N/A	N/A	N/A
Ceftiamidine	Not Included	4	1.9 (5.1)	5	2.2 (5.9)	7	2 (4.3)	N/A	N/A	N/A	N/A	N/A	N/A	N/A	N/A	N/A	N/A	N/A	N/A	N/A	N/A	N/A	N/A
Cefpiramide	Watch	N/A	N/A	2	2.5 (6.7)	N/A	N/A	N/A	N/A	N/A	N/A	N/A	N/A	N/A	N/A	N/A	N/A	N/A	N/A	N/A	N/A	N/A	N/A

AWaRe: Access, Watch, Reserve.
N/A: not applicable.

Table S6. Access-to-Watch ratio results by hospital level in Xinjiang, 2012-2022.

Hospital level	Access-to-Watch ratio											AAPC, %	Lower 95% CI	Upper 95% CI	P-value
	Year														
	2012	2013	2014	2015	2016	2017	2018	2019	2020	2021	2022				
All hospitals	46.4%	40.4%	45.0%	44.4%	37.1%	35.2%	31.9%	28.5%	24.0%	26.5%	24.4%	-6.8	-8.4	-5.1	< 0.001***
Tertiary hospitals	47.1%	41.0%	46.9%	47.3%	38.0%	35.4%	33.0%	26.7%	21.4%	25.4%	24.0%	-7.9	-10.2	-5.5	< 0.001***
Secondary hospitals	40.3%	35.3%	36.1%	28.1%	32.8%	34.9%	29.0%	32.6%	30.3%	29.4%	25.2%	-3.1	-5.0	-1.2	0.006**

AAPC: Average Annual Percent Change and CI: confidence interval.
* p<0.05, ** p<0.01, *** p<0.001

Table S7. Comparison results between tertiary and secondary hospitals.

Antibiotic Classification	Hospital level	Rate of antibiotic use by inpatients defined as daily doses per 100 patient days (% of total antibiotic use)											AAPC, %	Lower 95% CI	Upper 95% CI	P-value for trend	P-value for parallel pairwise comparison	P-value for coincidence pairwise comparison
		Year																
		2012	2013	2014	2015	2016	2017	2018	2019	2020	2021	2022						
Total antibiotic use	Tertiary hospitals	36.8(100.0)	36.4(100.0)	35.0(100.0)	39.1(100.0)	39.4(100.0)	30.9(100.0)	37.4(100.0)	35.9(100.0)	34.1(100.0)	36.5(100.0)	27.2(100.0)	-1.6	-3.7	0.5	0.111	0.206	0.381
	Secondary hospitals	37.8(100.0)	37.2(100.0)	45.5(100.0)	33.6(100.0)	32.9(100.0)	35.2(100.0)	35.8(100.0)	33.1(100.0)	28.1(100.0)	30.4(100.0)	28.5(100.0)	-3.2	-5.1	-1.4	0.004**		
J01A	Tertiary hospitals	0.1(0.4)	0.1(0.4)	1.3(3.7)	2.8(7.0)	1.1(2.7)	0.8(2.7)	1.3(3.5)	0.9(2.6)	0.8(2.4)	2.3(6.2)	1.2(4.3)	30.1	-3.0	74.4	0.079	0.213	0.002**
	Secondary hospitals	0.0(0.1)	0.0(0.1)	0.0(0.1)	0.0(0.0)	0.0(0.0)	0.1(0.2)	0.0(0.1)	0.1(0.2)	0.0(0.1)	0.0(0.1)	0.4(1.4)	17.6	-6.2	47.6	0.140		
J01C	Tertiary hospitals	5.0(13.6)	5.3(14.6)	5.1(14.6)	5.2(13.3)	5.9(15)	4.0(12.9)	4.7(12.5)	4.2(11.7)	3.5(10.4)	4.1(11.3)	3.7(13.6)	-3.9	-6.1	-1.6	0.004**	0.712	0.406
	Secondary hospitals	4.6(12.3)	5.5(14.9)	6.7(14.7)	4.9(14.7)	5.0(15.2)	5.5(15.6)	5.2(14.4)	5.0(15.2)	3.6(13.0)	4.2(13.8)	4.0(14.1)	-1.9	-8.1	4.6	0.553		
J01D	Tertiary hospitals	19.9(54.0)	19.5(53.5)	18.0(51.4)	20.0(51.2)	19.9(50.4)	17.3(56.0)	19.5(52.0)	19.7(55.1)	20.0(58.7)	19.0(52.1)	14.2(52.3)	-2.4	-5.8	1.1	0.180	0.262	0.004**
	Secondary hospitals	23.2(61.6)	23.5(63.1)	26.7(58.6)	21.0(62.5)	20.4(61.9)	23.4(66.5)	23.6(65.9)	21.8(65.8)	19.0(67.8)	20.1(66.3)	17.7(62.0)	-2.5	-4.3	-0.7	0.013*		
J01E	Tertiary hospitals	0.1(0.2)	0.1(0.3)	0.4(1.0)	0.2(0.6)	0.2(0.5)	0.2(0.5)	0.4(1.0)	0.2(0.6)	0.1(0.4)	0.2(0.6)	0.2(0.6)	10.1	-8.9	33.1	0.321	0.545	0.003**
	Secondary hospitals	0.5(1.4)	0.0(0.0)	0.0(0.0)	0.0(0.0)	0.0(0.0)	0.0(0.0)	0.0(0.0)	0.0(0.0)	0.0(0.0)	0.0(0.0)	0.0(0.0)	-22.6	-33.4	-10.0	0.001**		
J01F	Tertiary hospitals	3.6(9.7)	3.2(8.9)	3.2(9.2)	3.5(8.9)	4.4(11.2)	3.2(10.3)	3.3(8.8)	2.9(8.2)	2.3(6.8)	3.9(10.6)	2.4(8.9)	-2.6	-6.2	1.1	0.145	0.008**	0.012*
	Secondary hospitals	3.4(9.0)	3.4(9.3)	5.2(11.3)	3.1(9.2)	3.1(9.4)	2.1(6.1)	2.3(6.3)	2.0(6.0)	1.5(5.2)	1.8(5.9)	1.8(6.5)	-9.2	-13.1	-5.2	0.001**		
J01G	Tertiary hospitals	1.9(5.3)	1.3(3.5)	0.8(2.3)	0.6(1.6)	0.7(1.7)	0.6(1.9)	0.6(1.6)	0.4(1.2)	0.4(1.2)	0.3(0.8)	0.2(0.7)	-17.0	-20.5	-13.5	< 0.001***	0.725	0.002**
	Secondary hospitals	0.9(2.5)	0.6(1.5)	0.8(1.7)	0.3(1.0)	0.2(0.7)	0.5(1.3)	0.4(1.2)	0.2(0.6)	0.1(0.4)	0.2(0.5)	0.1(0.4)	-17.9	-24.2	-11.2	< 0.001***		
J01M	Tertiary hospitals	4.3(11.6)	4.9(13.5)	4.5(12.8)	5.0(12.8)	5.6(14.3)	3.5(11.2)	6.2(16.6)	6.0(16.8)	5.3(15.6)	5.2(14.2)	4.1(15.0)	0.8	-3.1	4.9	0.650	0.959	0.002**

	Secondary hospitals	3.1(8.2)	2.7(7.4)	4.1(9.0)	3.3(9.8)	3(9.2)	2.2(6.3)	3.2(8.9)	3.2(9.7)	3.1(10.9)	3.3(11.0)	3.7(13.0)	0.7	-2.8	4.3	0.670		
J01X	Tertiary hospitals	1.9(5.2)	1.9(5.4)	1.7(5.0)	1.7(4.4)	1.7(4.2)	1.4(4.4)	1.5(4.0)	1.3(3.7)	1.5(4.5)	1.5(4.2)	1.3(4.7)	-3.6	-5.2	-1.9	0.001**	0.046*	0.012*
	Secondary hospitals	1.9(5.0)	1.4(3.7)	2.1(4.6)	0.9(2.8)	1.2(3.6)	1.4(4.0)	1.1(3.1)	0.8(2.6)	0.8(2.7)	0.7(2.3)	0.7(2.5)	-9.3	-13.2	-5.3	0.001**		
Access	Tertiary hospitals	10.1(27.3)	8.9(24.5)	9.4(26.7)	10.4(26.5)	9.2(23.3)	6.7(21.7)	8.0(21.5)	6.5(18.0)	5.1(15.0)	6.4(17.7)	4.5(16.6)	-7.2	-9.9	-4.4	< 0.001***	0.100	**0.313**
	Secondary hospitals	9.5(25.2)	8.5(22.9)	10.5(23.0)	6.3(18.6)	7.1(21.6)	7.7(22.0)	7.0(19.7)	7.3(22.1)	5.9(20.9)	6.4(21.0)	5.2(18.3)	-5.0	-7.5	-2.3	0.002**		
Watch	Tertiary hospitals	21.4(57.9)	21.7(59.7)	20(57.0)	21.9(56.1)	24.2(61.4)	18.9(61.3)	24.4(65.1)	24.1(67.3)	23.9(70.2)	25.4(69.6)	18.8(69.2)	0.7	-1.7	3.1	0.539	0.066	**0.179**
	Secondary hospitals	23.6(62.6)	24.1(64.8)	29.0(63.7)	22.3(66.3)	21.7(65.8)	22.2(62.9)	24.3(67.7)	22.4(67.6)	19.4(68.9)	21.7(71.4)	20.7(72.5)	-2.0	-3.7	-0.1	0.038*		
Reserve	Tertiary hospitals	0.6(1.6)	0.4(1.1)	0.3(0.8)	0.3(0.9)	0.4(0.9)	0.3(0.8)	0.3(0.9)	0.5(1.3)	0.7(1.9)	0.8(2.2)	0.7(2.6)	6.2	-3.4	16.6	0.213	0.228	**0.002** **
	Secondary hospitals	0.7(1.9)	0.1(0.2)	0.0(0.1)	0.0(0.0)	0.0(0.1)	0.1(0.3)	0.1(0.2)	0.0(0.1)	0.0(0.1)	0.0(0.1)	0.1(0.3)	-20.8	-47.7	19.9	0.270		
Not recommended	Tertiary hospitals	2.4(6.6)	3.4(9.3)	3.8(10.9)	5(12.7)	4.4(11.2)	4.1(13.4)	4.2(11.2)	4.7(13.0)	4.3(12.7)	3.7(10.1)	3.0(11.1)	3.2	-2.1	8.8	0.246	0.378	**0.017** *
	Secondary hospitals	1.9(4.9)	2.3(6.1)	3.6(7.8)	3.9(11.7)	3.4(10.3)	4.2(12.0)	4.0(11.1)	3.2(9.7)	2.8(10.0)	2.3(7.5)	2.5(8.9)	2.9	-3.7	10.0	0.395		
Not included	Tertiary hospitals	2.4(6.5)	2.0(5.4)	1.6(4.6)	1.5(3.8)	1.2(3.2)	0.9(2.8)	0.5(1.3)	0.2(0.5)	0.1(0.2)	0.2(0.5)	0.1(0.4)	-30.3	-37.6	-22.1	< 0.001***	0.010*	**0.197**
	Secondary hospitals	2.0(5.4)	2.3(6.1)	2.5(5.5)	1.1(3.3)	0.7(2.1)	1.0(2.9)	0.5(1.3)	0.2(0.5)	0.0(0.1)	0.0(0.0)	0.0(0.0)	-45.1	-57.6	-28.8	< 0.001***		

Note: The value of 0.0 in the table does not indicate an actual value of zero but represents a number that has been rounded to one decimal place, with subsequent decimal places not visible due to rounding.

AAPC: Average Annual Percent Change and CI: confidence interval.

* p<0.05, ** p<0.01, and *** p<0.001
